# Low apparent diffusion coefficient in an extracranial brain tumor: a case report

**DOI:** 10.1186/s13256-022-03553-x

**Published:** 2022-08-19

**Authors:** Ho Xuan Tuan, Nguyen Duy Hung, Nguyen Dinh Minh, Nguyen Nhat Quang, Ngo Quang Duy, Nguyen Minh Duc

**Affiliations:** 1 Department of Medical Imaging, University of Medical Technology and Pharmacy, Da Nang, 500000 Vietnam; 2 Department of Radiology, Hanoi Medical University, Hanoi, 100000 Vietnam; 3 Department of Radiology, Viet Duc Hospital, Hanoi, 100000 Vietnam; 4 Department of Radiology, Ha Giang General Hospital, Ha Giang, 200000 Vietnam; 5 Department of Radiology, Pham Ngoc Thach University of Medicine, 2 Duong Quang Trung, Ward 12, District 10, Ho Chi Minh City, 700000 Vietnam

**Keywords:** Apparent diffusion coefficient, Case report, Extra-axial, Magnetic resonance imaging, Medulloblastoma

## Abstract

**Background:**

Medulloblastoma is well known as the most common malignant brain tumor identified in children, frequently found at an intra-axial location in the posterior cranial fossa. Extra-axial medulloblastoma is uncommon and often misdiagnosed. We believe that a thorough understanding of atypical medulloblastoma cases is important in daily practice.

**Case presentation:**

We present the unique case of a 39-year-old woman of Asian descent who suffered from headaches and right-sided hearing impairment. A right extra-axial medulloblastoma with an extremely low apparent diffusion coefficient of 0.404 × 10^−3^ mm^2^/second was detected on magnetic resonance imaging. The initial diagnosis suggested schwannoma or hemangioblastoma. However, the postoperative histopathologic findings indicated medulloblastoma (World Health Organization grade IV). Pre- and postoperative magnetic resonance imaging revealed no drop metastasis, but adjuvant radiation therapy was still required as a standard treatment therapy

**Conclusions:**

Extra-axial medulloblastoma is an uncommon tumor that is often mistaken for other cerebellopontine angle neoplasms. We describe a rare example of extra-axial medulloblastoma, characterized by a low apparent diffusion coefficient. When evaluating an atypical cerebellopontine angle neoplasm, the apparent diffusion coefficient should be considered a relevant indicator.

## Introduction

At the most basic histological level, the human brain consists primarily of two components: neuronal cells and glial cells. Glial cells are typically viewed as supportive cells able to undergo cell division to replenish themselves after injury. By contrast, neuronal cells are more stable and often do not divide after differentiation. Therefore, the vast majority of brain tumors have glial cell components; medulloblastoma is an unusual exception owing to its neuronal cell origins. Medulloblastoma is thought to derive from primitive neuronal cells and is expected to be found in intra-axial locations of the brain and spine [1]. On extremely rare occasions, medulloblastoma can be observed in an extra-axial location: the cerebellopontine angle (CPA) (86%), tentorial membrane (8%), or lateral hemispheric region of the cerebellum (6%) [1, 2].

Clinical symptoms also vary on the basis of location, and patients often complain of headaches and persistent vomiting [3]. Truncal ataxia and spasticity are often indicators of tumors located in the vermis, whereas nystagmus and limb ataxia predominantly suggest a hemispheric location. Abducens nerve palsy may result from an extraventricular medulloblastoma extension [4]. On radiographic examination, the most distinctive features used to distinguish medulloblastoma from other glial tumors are hyperdensity on computed tomography (CT) and restricted diffusion on magnetic resonance imaging (MRI), which are attributed to the high cellularity of medulloblastoma [1]. Although this neoplasm can present in both intra- and extra-axial locations, a very low apparent diffusion coefficient (ADC), to our knowledge, is unique to medulloblastoma [1]. We present the case of a 39-year-old woman who suffered from an extra-axial medulloblastoma with a remarkably low ADC located in the right CPA and retrospectively analyzed several reported cases of extra-axial medulloblastoma.

## Case

A 39-year-old Asian woman presented to our hospital with symptoms including headache, dizziness, and right-sided hearing impairment, which she had experienced for some time but described as worsening starting 2 months prior to presentation. The patient did not adopt any specific medications until hospital admission. She was a multi-para woman (gravida 2, para 2, living 2, abortion 0). The family members did not have any specific diseases and did not suffer from the same symptoms. She lives in Hanoi and worked as an accountant with normal medical profile prior to this admission. On physical examination, the patient’s weight and height were 50 kg and 162 cm, respectively [body mass index (BMI) of 19]. Vital signs were within normal range, with body temperature of 37 °C, pulse rate of 80 beats per minute, blood pressure of 120/70 mmHg, and respiratory rate of 16 breaths per minute. Upon neurological examination, no neurological defects of sensorium, cognition, cranial nerves, motor, sensory, cerebellar, gait, reflexes, meningeal irritation, or long tract signs were observed. Blood test findings were normal in her report (glucose of 80 mg/dl, blood urea nitrogen of 12 mg/dl, creatinine of 1 mg/dl, alanine aminotransferase of 22 U/L, aspartate aminotransferase of 32 U/L, white blood cell count of 7.8 × 10^9^/L, red blood cell count of 5 × 10^9^/L, hemoglobin of 12.8 g/dl, and hematocrit of 42%).

**Fig. 1:**
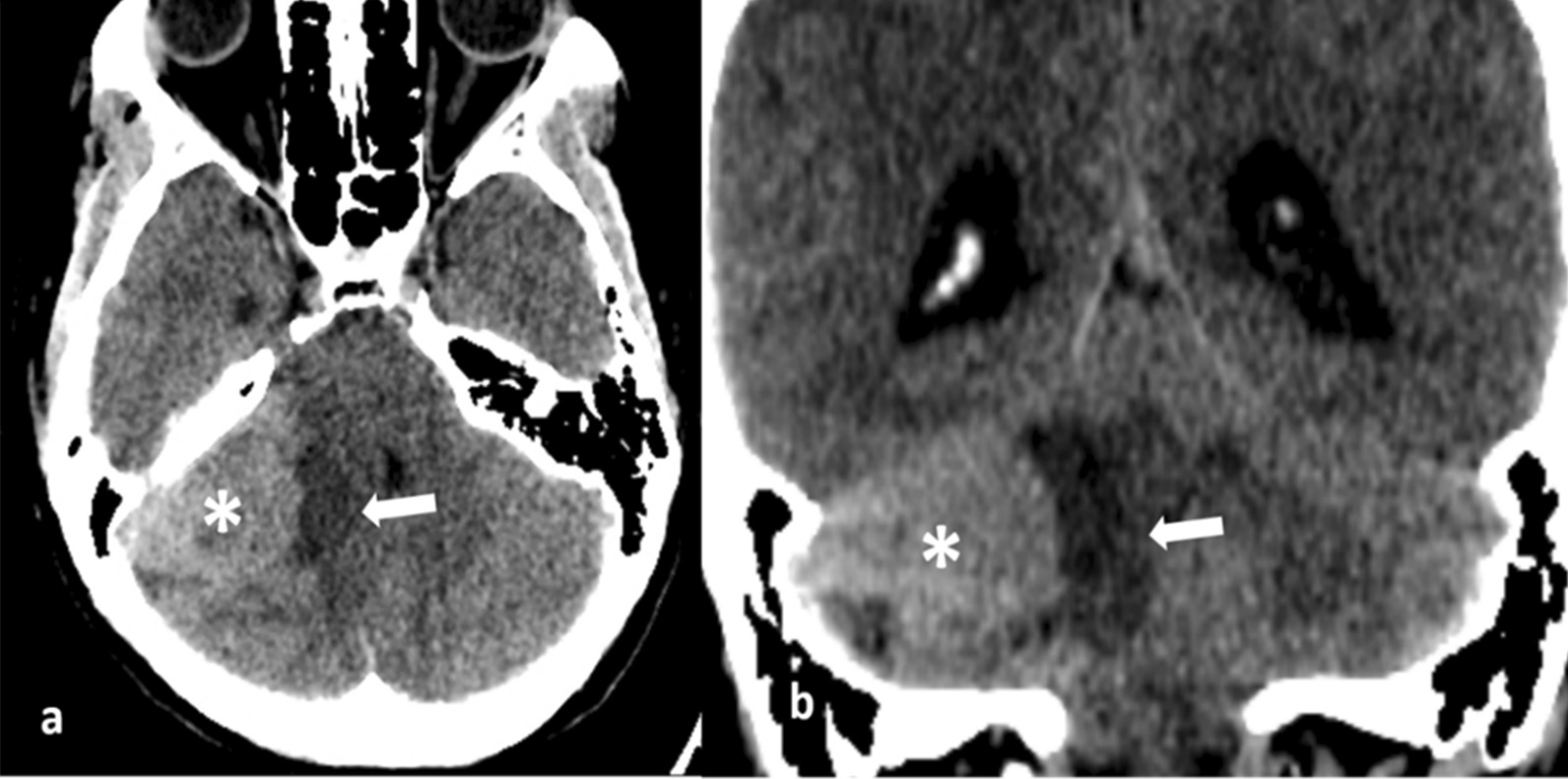
Nonenhanced computed tomography image on the axial plane (**a**) and coronal plane (**b**) showing a right cerebellopontine angle mass (asterisk) that is hyperdense compared with normal tissues. Moderate cerebral edema surrounding the mass can be observed (arrow)

On head CT, we detected a 50 × 40 × 28 mm mass in the right CPA primarily consisting of a solid component with slight hyperdensity and some cystic-like proximal structures. No calcification or hemorrhage was identified. A mass effect could be observed in the fourth ventricle and cerebellar peduncle, which dilated the ventricular system upstream (Fig. 1).

**Fig. 2:**
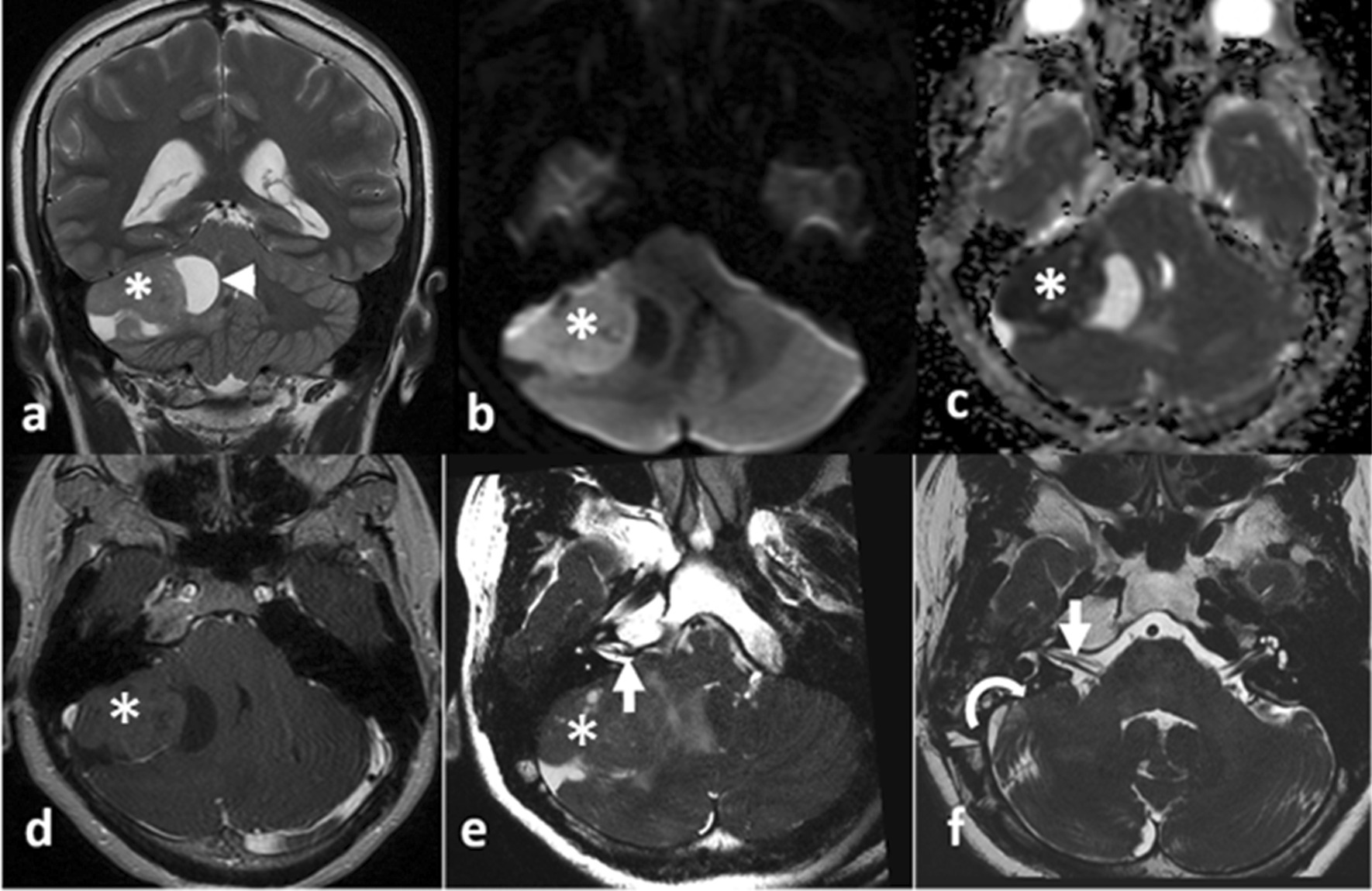
Magnetic resonance imaging from the preoperative examination (**a**–**e**) and 3 weeks after the operation (**f**). A mixed-signal mass (asterisk) was observed in the right cerebellopontine angle, presenting with solid and cystic components (white arrowhead) on T2-weighted coronal imaging (**a**). The solid component showed hypersignal on diffusion-weighted imaging (**b**) and significant hyposignal on the apparent diffusion coefficient map (**c**). After contrast injection, this mass was poorly enhanced (**d**). On the 3D-constructive interference in steady-state sequence (**e**), the VII/VIII cranial nerve complex (white arrow) was observed to be anteriorly displaced. The postoperative axial 3D-constructive interference in steady-state sequence (**f**) revealed a normal right cerebellar hemisphere (white curved arrow), and the VII/VIII cranial nerve complex (white arrow) returned to its expected location

On the following day, MRI was performed. A lobulated mass on the right tentorium showed mixed-signal features, including hypersignal on T2-weighted image (T2WI) and fluid-attenuated inversion recovery. A very low ADC of 0.404 × 10^−3^ mm^2^/second was observed, corresponding with the hyperdense feature on CT, indicating a suspected high-cellularity neoplasm. In the post-contrast sequence, poor enhancement characteristics and some small blood vessels were documented. A schwannoma case was suspected, and a three-dimensional constructive interference in steady-state (3D-CISS) sequence was ordered, which revealed the preservation of the right internal acoustic meatus with slightly anteriorly displaced nerves (Fig. 2). The 3D-CISS results allowed for the exclusion of schwannoma, indicating a probable diagnosis of hemangioblastoma.

The patient underwent a retrosigmoidal suboccipital craniectomy for tumor resection. According to the operative report, this CPA tumor was located close to the dural matter and was soft with hypervascularity. The preoperative specimen confirmed a medulloblastoma case, and gross total excision was performed.

**Fig. 3:**
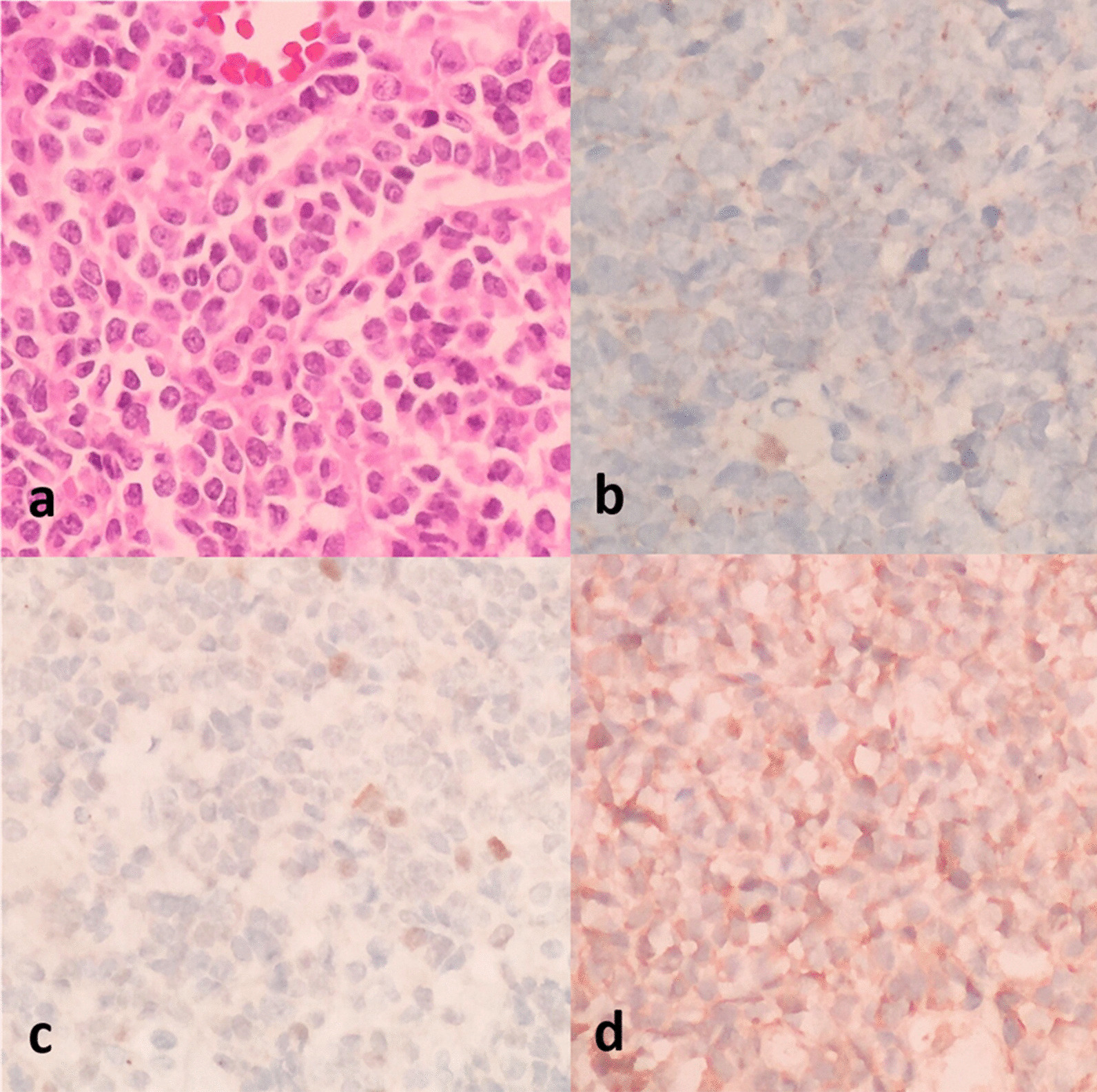
Histopathologic results: (**a**) a hypercellular mass with carrot-shaped, dense nuclei. Immunohistochemical stains were negative for beta-catenin (**b**) and p53 (**c**) and positive for synaptophysin (**d**)

The postoperative histopathological result revealed that this neoplasm was composed of multiple cells with carrot-shaped nuclei and scant cytoplasm. The tumor cells were arranged in sheets, rows, or nodules. Immunohistochemical stains were positive for oligodendrocyte transcription factor 2, synaptophysin, and INI1 but negative for glial fibrillary acidic protein, beta-catenin, and p53 (Fig. 3). These pathological findings are consistent with a medulloblastoma (World Health Organization grade IV) diagnosis.

Three weeks after surgery, the patient returned for a postoperative follow-up. The brain MRI and whole spine scan detected no evidence of recurrence or metastatic signs, respectively. She received adjuvant radiation therapy in keeping with our standard of care for this type of tumor. After 6 months of follow-up, the patient did not suffer from any adverse events and the symptoms were completely resolved.

## Discussion

In this article, we described the case of a 39-year-old lady who had an extra-axial medulloblastoma with a surprisingly low ADC that was in the right CPA. The term “extra-axial medulloblastoma” is used to describe any tumor that arises external to the brain parenchyma (that is, in the bone, meningeal membrane, nerve, accessory parts) [5]. The mechanism underlying EAM formation has not yet been established, and several theories have been posited to explain how these tumors develop. One theory suggests that these tumors arise from the external granular cell layer, migrating from the posterior medullary velum or cerebellar flocculus [6]. Another theory suggests that extra-axial medulloblastomas located in the CPA originate as exophytic cerebellar or pons medulloblastomas that spread laterally through the foramen of Luschka to the CPA region [7].

The clinical symptoms associated with medulloblastoma are often nonspecific, but some hallmarks can be used for locational diagnosis. In our case, long-lasting hearing impairments were significant indicators of a CPA lesion affecting cranial nerve VIII. CPA neoplasms represent approximately 10% of all intracranial tumors [8]. Vestibular schwannoma accounts for 75–80% of all CPA tumors, whereas meningioma accounts for 10–15% of CPA tumors, epidermoid cysts represent 7–8%, and all other tumor types are identified in only 1% of cases [8, 9]. Therefore, we highly recommend the 3D-CISS sequence as a useful tool for vestibular nerve evaluation in all CPA lesions. Some studies suggest that timing may contribute to differential diagnoses between intra-axial and extra-axial CPA lesions. An intra-axial lesion should present as a rapidly progressing neoplasm, and brainstem dysfunction and hydrocephalus are expected to appear soon after the initial symptoms, rather than presenting with slow progression as observed in our case [10].

The differentiation between CPA medulloblastoma and other CPA tumors can be extremely difficult [9, 11]. In 2009, Furtado *et al*. reported a CPA medulloblastoma case with a “dural tail sign” and hyperostosis, which mimicked a posterior petrous meningioma [12]. Cystic changes or calcification features have also been described in cases of CPA medulloblastoma. Our case exhibited features similar to the case described by Yamada *et al*. [7]. Hemangioblastoma was diagnosed according to several reasonable characteristics. However, even though hemangioblastoma was the most likely diagnosis, some inconsistencies were noted. Traditionally, the solid component of hemangioblastoma is described as a mural nodule, with vivid enhancement during the post-contrast phase and often characterized by multiple internal flow void components. By contrast, our patient’s neoplasm was poorly enhanced, with no convincing flow voids identified on T2WI. In addition, we detected an abnormally low ADC for this tumor, with a mean value of only 0.404 × 10^−3^ mm^2^/second. In 2020, Mert Doğan *et al*. described a study of 56 pediatric patients with brain tumor. The mean ADC for hemangioblastoma was reported as 1.72 ± 0.25 × 10^−3^ mm^2^/second, which is significantly lower than the ADC for medulloblastoma (0.63 ± 0.22 × 10^−3^ mm^2^/second), and the cutoff value they suggested for differentiating between low- and high-grade pediatric brain tumors was 1.1 × 10^−3^ mm^2^/second [13]. In a previous report, a CPA medulloblastoma had a very low ADC of 0.4 × 10^−3^ mm^2^/second [14]. Our findings were in full agreement with this report.

Treatment options include observation, radiation therapy, and microsurgery. Observation was not considered in our case owing to the patient’s young age and cranial nerve function problems, which can typically be addressed through microsurgery. Several microsurgery approaches have been introduced [15], and the middle cranial fossa approach is the most commonly used approach in cases with intracanalicular extensions. The translabyrinthine approach can be useful in patients without any serviceable hearing [16]. Our patient was most ideally suited for the suboccipital and retrosigmoidal approach, which is less invasive and more likely to preserve hearing. However, the high risk of recurrence should be monitored during postoperative management [15, 17]. Owing to the high prevalence of cerebral spinal fluid seeding associated with medulloblastoma, pre- and postoperative whole spine imaging studies are usually performed. Drop metastasis due to leptomeningeal seeding should be carefully excluded in all situations. Fortunately, our patient presented with no evidence of recurrence 3 weeks after the operation. Therefore, adjuvant radiation therapy was the most suitable postoperative approach.

The recurrence rate of adult medulloblastoma is roughly 50–60% [18]. In contrast to children, adult medulloblastoma recurrence often occurs late, with one recurrence case reported 14 years after treatment [19]. Thus, long-term follow-up is necessary after surgery for the best outcome.

## Conclusion

Extra-axial medulloblastoma is a rare entity that can easily be misdiagnosed as other CPA tumors. We report a rare case of extra-axial medulloblastoma characterized by low ADC. ADC should be carefully considered for all atypical CPA neoplasms.

## Data Availability

Not applicable. All data requests should be submitted to the corresponding author for consideration. Access to anonymized data may be granted following review.
